# Effects of volcanic eruptions on the mental health of exposed populations: a systematic review

**DOI:** 10.3389/fpubh.2024.1475459

**Published:** 2024-12-12

**Authors:** Danae A. Mendoza, José M. Reales, Soledad Ballesteros

**Affiliations:** 1 Doctoral School Universidad Nacional de Educación a Distancia (UNED), Madrid, Spain; 2 Department Methodology of Behavioral Sciences, Facultad de Psicología, Universidad Nacional a Distancia (UNED), Madrid, Spain; 3 Department of Basic Psychology II, Facultad de Psicología, Universidad Nacional de Educación a Distancia (UNED), Madrid, Spain

**Keywords:** anxiety, depression, post-traumatic stress disorder (PTSD), stress, volcano, volcanic eruption

## Abstract

**Introduction:**

Millions of people living in volcanic environments are at risk of experiencing volcanic eruptions, a natural disaster. This systematic review aimed to collect empirical evidence of the effects of volcanic eruptions on the mental health of the exposed populations.

**Methods:**

Following the Preferred Reporting Items for Systematic Reviews and Meta-Analyses (PRISMA) guidelines, we conducted systematic searches on Scopus, PubMed, PsycINFO, Medline, and Web of Science (WoS) databases.

**Results:**

A total of 17,044 articles were screened. Of these, 24 articles met the inclusion criteria and data were extracted. Twenty-one articles investigated psychological disorders, two articles studied emotions and their relationships with certain environmental factors, and one article explored cognitive functions in the exposed populations. These studies showed that highly exposed populations were more vulnerable to develop long-lasting psychological disorders than less exposed populations.

**Conclusion:**

The negative influence of experiencing volcanic activity on mental health was confirmed. Clearly, there is a need for more research to improve the mental health of the populations highly exposed to volcanic eruptions. Recommendations for future research are also included.

## 1 Introduction

As the world’s population continues to grow, the number of people living in the vicinity of volcanoes has increased proportionally (1). Approximately 22 million people currently live within a 5 km radius of active volcanoes [calculations based on data provided by the Smithsonian Institution (2)] and are fully exposed to a possible volcanic eruption.

Volcanic eruptions produce dangerous effects on the environment, climate, and the health of exposed people by expelling magma, steam (H_2_O), gases (CO_2_, SO_2_, CO, H_2_S, CS, CS_2_, HCI, H_2_, CH_4_, HF, HBr), organic compounds, and heavy metals such as mercury, lead, and gold (3).

Evidence from a systematic review (4) suggests that respiratory complications due to volcanic eruptions are the most common in acute symptoms (e.g., asthma, bronchitis) especially in individuals with pre-existing lung disease. Chronic symptoms include asbestosis, silicosis, and mesothelioma produced by volcanic ash. This refers strictly to all particles ejected by a volcano that are ≤ 2 mm in diameter, regardless of size, composition, shape, and density (5).

The accumulation of these volcanic debris in the long run can produce contamination of the nearby areas of the volcano, resulting in damage to the health of exposed living beings. Findings showed an accumulation of fluoride (F) in cow rib bone and teeth in grazing animals, which probably consume F-bearing volcanic ash and gas hydrates on the surface of plant leaves and in drinking water at higher levels than those recommended by the World Health Organization (WHO) (6).

The relationship between volcanic eruption in the exposed population and the development of poor mental health has been investigated for more than 30 years. However, as far as we know, no systematic review has examined the exclusive influence of volcanic eruptions on the mental health of the exposed populations. Instead, there are reviews on the negative effects on the mental health of survivors of another natural disaster such as earthquakes, floods, etc., which do not include volcanic eruptions. The findings indicate that post-traumatic stress disorder (PTSD) is the most persistent psychological disorder after natural disasters, followed by anxiety, depression, and other behavioural disorders, as well as psychological abnormalities (7). Specific populations appear to be particularly vulnerable to experiencing mental health disorders following a natural disaster. For example, in women, a significant correlation was found between mental health after experiencing a natural disaster and increased exposure to domestic violence (8). Older adults exposed to natural disasters are 2.11 times more likely to experience PTSD symptoms and 1.73 more likely to develop adjustment disorder compared with younger adults (9). Likewise, those who suffer from low social support and parentless children were highly vulnerable to suicide after suffering natural disasters (10).

This lack of information about volcanic eruption survivors is also found in cognitive impairment. Along these lines, the literature on cognitive functions in other natural disasters is also limited and heterogeneous. A meta-analysis of fMRI studies of PTSD in survivors of natural disasters showed activation foci in the superior and inferior frontal gyrus, insula and lingual gyrus in the right hemisphere, and this neuro-functional alteration suggests the presence of selective cognitive deficits in visuospatial and navigational memory (11). Among Hurricane Katrina survivors, it was observed that when symptoms of depression and PTSD were controlled, only deficits in attention were independent of emotional symptoms (12). Likewise, in the sustained-attention-to-response task, an increase in errors of omission was found, but errors of commission and reaction times were dependent on individual differences in stress response to the earthquake (13). In older adults, cognitive deficits associated with natural disasters are especially remarkable. For example, living at home (14) during floods, or home damage (15, 16) and housing loss (17) due to earthquakes and tsunamis, were associated with an increased risk of cognitive decline (e.g., short-term memory, orientation, and communication), while informal socialization and social participation had a protective effect in the incidence of cognitive decline (15, 16) after earthquake and tsunami.

Volcanic eruptions are characterized by episodes of an indeterminate period with variable magnitude. In the actual climate change scenario, the contribution of volcanic eruptions and their effects on climate change are underestimated. Recently, a simulation on standard climate projections by Chim et al. (18) found that small-magnitude eruptions contribute between 33% and 40% of total volcanic SO2 emissions and, in turn, are responsible for 30 to 50% of the impact on large-scale climate indicators of global surface temperature, sea level, and sea ice extent.

This underestimation of volcanic eruptions and their effects is reflected also in the field of environmental psychology and ecopsychology. In particular, in the construct of climate emotions (also known as eco-emotions), where models such as Doherty and Clayton’s (19) or recent systematic reviews on the impact of climate change on mental health (20, 21) do not consider volcanoes among the natural disasters listed as potential contributors to climate change. However, Warsini et al. (22) argued that solastalgia, a term created by Albrecht (23) composed of concepts “solace” and “desolation” that describes the pain or sickness caused by the loss or lack of solace and the sense of isolation connected to the present state of one’s home and territory, can be studied among people who have been exposed to volcanic eruptions because these people had a loss or a potential loss of place, identity, and previous comfort due to the environmental damage caused by the volcano.

## 2 Research aims

To bridge the gap in the literature, this systematic review examines the effects on mental health in populations who have experienced or are currently experiencing a volcanic eruption or its aftermath.

The question underlying this review is: *what effects have volcanic eruptions had on the mental health of people of all ages who have experienced this natural disaster?* To answer this, four aims were proposed: (1) to investigate the most common psychological disorders reported in quantitative studies conducted on children, adults and older adults who have experienced or are experiencing a volcanic eruption; (2) to explore the effects of volcanic eruptions on cognitive functioning; (3) to investigate the presence of emotions and eco-emotions reported in quantitative studies; and (4) to identify possible novel factors and limitations in the existing literature.

In the following sections, we present the search strategy used for this review, the inclusion and exclusion criteria, the main findings yielded by the reviewed articles and the implications for future research.

## 3 Methods

### 3.1 Inclusion and exclusion criteria

Studies in this systematic review were included if they met the following inclusion criteria: (1) the articles were written in English; (2) they were published in peer-reviewed journals; (3) participants had experienced a volcanic eruption; (4) the article reported quantitative data; and (5) the study provided at least an outcome of mental health or cognitive functioning of people who have experienced a volcanic eruption without age restrictions; finally, (6) the studies included in this review were published from inception to October 2023.

Studies were excluded if they were not published in English in peer-reviewed journals (e.g., opinion, editorials, grey literature, reports); they presented only qualitative data; the article did not show primary or original data (e.g., reviews, editorials, conferences papers, abstracts congress, protocols); or if the article did not focus on mental health or cognitive functions.

### 3.2 Search strategy

The current systematic review followed the Preferred Reporting Items for Systematic Reviews and Meta-analysis (PRISMA and PRISMA-P) statement (24, 25). We performed systematic electronic searches at Scopus, PubMed, WoS, Medline and PsycINFO databases on 23 October 2023. These databases were selected because they are the most widely used to ensure that relevant publications were not missed and consulting with librarians. No date limitations were imposed in the searches.

The search terms were intersections referring to the combination of terms associated with volcanoes and terms denoting mental health, such as the possible psychological disorders or cognitive impairments found among people who have experienced a volcanic eruption. Notwithstanding, the search formula was broad enough to avoid restrictions on the results, using the following string:

(“volcanic eruption” OR volcano OR “volcanic disaster”) AND (“mental health” OR “mental disorder” OR “mental effect” OR “mental symptoms” OR “psychological health” OR “psychological disorder” OR “psychological effect” OR “psychological symptoms” OR psychopatholog* OR pyscholog* OR emotion* OR eco-emotion OR PTSD OR “post-traumatic stress disorder” OR anxiety OR depression OR stress OR “cognitive impairments” OR “psychological impairments” OR cogniti* OR brain OR executive OR attention* OR memor*).

### 3.3 Selection process

**Figure 1:**
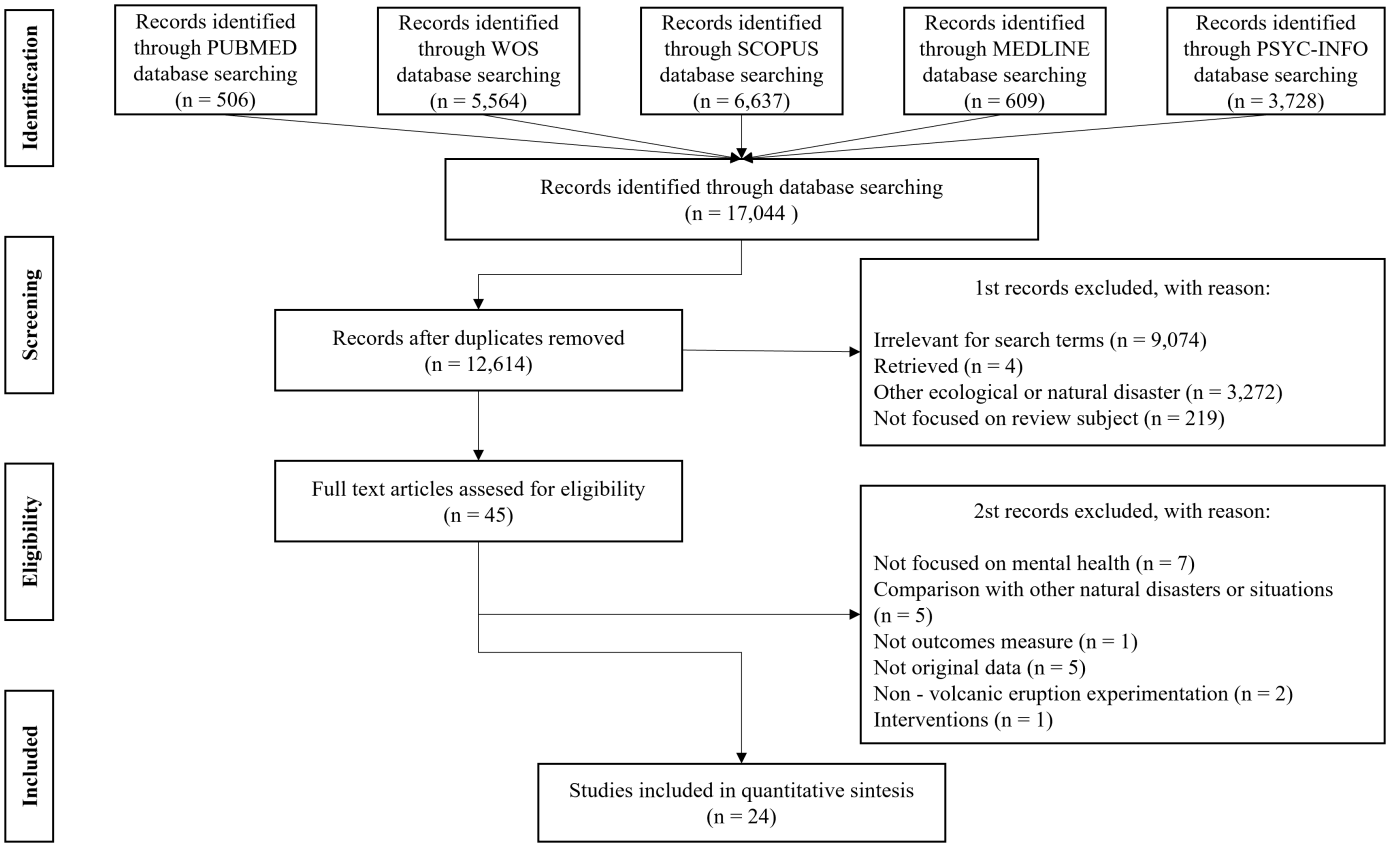
PRISMA flowchart diagram of the search strategy.

The search in the databases yielded a total of 17,044 articles, but this number was significantly reduced to 12,614 articles after removing duplicates. The results were meticulously reviewed by two of the authors (DM and JR), first reading the titles and abstracts of the retrieved studies to remove articles that clearly did not comply with the selection criteria. After this initial evaluation, 45 potential articles were identified for full reading and further assessment. To ensure internal validity within the review, all studies that were considered relevant were read in full and independently by two of the authors for their inclusion. When there was a disagreement about the inclusion of a certain study, the matter was discussed with the third author (SB) until they reached an agreement. Finally, 24 articles that met the inclusion criteria were included in this review. Figure 1 shows the PRISMA flow diagram of the systematic search and study selection.

### 3.4 Data extraction and synthesis

Two authors carefully read and coded the data from the 24 included articles using the same codebook, and then a data extraction was performed to collect information relevant to the review question. Specifically, the data extracted from the eligible papers were the following: (1) sample characteristics (age, gender, country of origin, volcano, period since eruption); (2) details of study design; (3) outcome measures; and (4) significant findings.

Meta-analysis was inappropriate due to the high heterogeneity in the studies in terms of the samples, the lack of a control group or baseline measurement, the characteristics of each volcanic eruption, the period since the eruption, and the variety of data collection methods.

### 3.5 Quality appraisal

Risk of bias, an indicator of the methodological quality of the studies, was assessed using the Standard Quality Assessment Criteria for Evaluating Primary Research Papers from a Variety of Fields by Kmet et al. (26). This tool was selected because it is useful for evaluating quantitative and qualitative studies in a variety of fields. This checklist contains 14 quality criteria for assessing the risk of bias in the studies, namely: (1) Question/objective sufficiently described? (2) Study design evident and appropriate? (3) Method of subject/comparison group selection or source of information/input variables described and appropriate? (4) Subject (and comparison group, if applicable) characteristics sufficiently described? (5) If interventional and random allocation were possible, was it described? (6) If interventional and blinding of investigators was possible, was it reported? (7) If interventional and blinding of subjects was possible, was it reported? (8) Outcome and (if applicable) exposure measure(s) well defined and robust to measurement / misclassification bias? Means of assessment reported? (9) Sample size appropriate? (10) Analytic methods described/justified and appropriate? (11) Is some estimate of variance reported for the main results? (12) Controlled for confounding? (13) Results reported in sufficient detail? (14) Conclusions supported by the results?

Items are rated on a three-point scale (yes = 2, partial = 1 and no = 0), with a maximum score of 28 on the quantitative studies. Items 5, 6, and 7 did not qualify for particular study designs, were marked “N/A” and excluded from the total score calculation. There is no accepted cut-off for a quality rating of checklist scores. Therefore, no studies were excluded due to low quality. We used the categorization proposed by Van Cutsem et al. (27), which includes a score ≥ 75% indicated strong quality, 55–75% specified moderate quality, and a score ≤ 55% showed weak quality. The inter-judge agreement was calculated using quality scores the first two authors provided independently. Disagreements were resolved by consensus or consultation with the third author. For a detailed description of the quality assessment of the reviewed articles (see Supplementary Material S1).

## 4 Results

A total of 24 studies were deemed eligible. Table 1 shows the study characteristics of the cross-sectional studies, and Table 2 displays the characteristics of the longitudinal studies including: author(s), publication year, country, study’s design, sample, outcomes measures, main findings and the quality assessment.

**Table 1:** Characteristics of the cross-sectional studies included in this review.

First author, publication year	Country, volcano, period since eruption	Study design	Participants (*n*, mean age, % gender)	Outcomes measures	Significant findings	Quality assessment
Carlsen et al., 2012 (48)	Iceland, Eyjafjallajökull; 6–9 months	Cross-sectional; cohort; control group; exposure level groups	*n* = 1,658; Exposed *n* = 1,148 (Low expose *n* = 152; Medium *n* = 644; High expose *n* = 352); age range 18–80y; *F* = 51%; Non-exposed *n* = 510; age range 18–80y; *F* = 48.6%	GHQ-12	Psychological morbidity symptoms were found in the exposed population (OR 1.3; 95% CI 1.0 to 1.7; ≅d=0.14 ).	Strong
Carlsen et al., 2012 (45)	Iceland, Eyjafjallajökull; 6–9 weeks	Cross-sectional; no control group; no exposure level groups	*Adults: n* = 150; age range 18–>64y; F = not mentioned *Children: n* = 40; age range 0–17y; *F* = 53%	GHQ-12; DASS; PSS-SR	39% of the participants reported some symptoms of mental distress; less than 10% reported symptoms of stress, anxiety, depression, or PTSD. No effect size available.	Strong
Escolà-Gascón et al., 2023 (39)	Spain, Tajogaite; 1 year	Cross-sectional; control group; no exposure level groups	*n* = 502; mean age = 39.87y, age range 21–60y; *F* = 48%; Exposed *n* = 281; Non-exposed *n* = 221	NEP; Willingness to sacrifice for the environment; APAS; STAI	Volcanic eruption was predictor of reduction in pro-ecological attitudes (43.9%), in sense of place (36.8%) and stress levels (92.8%). NEP *d* effect sizes = 0.10, −0.73 and-0.00; APAS *d* effect sizes = −0.41, −0.00, and-0.79; STAI *d* effect sizes = 1.28, 2.04, and 1.44.	Strong
Gissurardóttir et al., 2019 (49)	Iceland, Eyjafjallajökull; 6–9 months	Cross-sectional; population based; no control group; exposure level groups	*n* = 1,656; Exposed *n* = 1,146 (Low Expose *n* = 152; age range 18–50y, *F* = 48.7%; High Exposure *n* = 994, age range 18–80y, *F* = 49.1%); age range 18-80y; F = 51%; Non-exposed *n* = 510; age range 18–80y; F = 48.6%	GHQ-12; PSS-4; PC-PTSD	High exposure group had an increased risk of experiencing mental distress (OR 1.45; 95% CI 1.11–1.90; ≅d=0.203 ) and PTSD symptoms (OR 3.71; 95% CI 1.34–15.41; ≅d=0.723 ) compared to the non-exposed group.	Strong
Goto et al., 2002 (40)	Japan, Miyake Island; 10 months	Cross-sectional; no control group; no exposure level groups	*n* = 231; mean age = 59.52y, age range 20–80+; *F* = 46.3%	IES-R; CES-D	Severity of PTSD and depression symptoms were positively correlated with help-seeking, from physicians, but not psychologist or mental health professionals. No effect size available.	Strong
Goto et al., 2006 (41)	Japan, Miyake Island; 10 months	Cross-sectional; no control group; no exposure levels groups	*n* = 231; mean age = 59.52y, age range 20–80 + y; F = 46.3%	IES-R; CES-D	PTSD symptoms were significantly associated to material loss and uncertainty of losses, multiple times reallocations and being older, widowed, lower SES, less education and longer length of residency. No effect size available.	Strong
Kamijo et al., 2020 (42)	Japan, Mt. Ontake; 1 year	Cross-sectional; no control group; no exposure levels groups	*n* = 213; age range 20–50 + y; *F* = 8.9%	PDS; CD-RISC	PTSD severity was associated with peritraumatic situation factors: female gender (OR 3.58, 95% CI 1.19–10.77; ≅d=0.703 ), cumulative days on duty ≥7 (OR 2.47; 95% CI 1.21–5.06; ≅d=0.498 ) and drinking or smoking as stress relief after disaster—support work (OR 2.35; 95% CI 1.09–5.04; ≅d=0.471 ).	Moderate
Kushnick et al., 2022 (31)	Indonesia, Sinabung; not mentioned	Cross-sectional; retrospective cohort; control group; no exposure levels groups	*n* = 194; Evacuees woman *n* = 97; mean age = 30.4y, age range 18–36 or older; Non-evacuees woman *n* = 97; mean age = 30.7y	IES-R	Birth length was lower with increasing stress related evacuation. IESR: Birth length: r=−0.27,p<.05 Birthweight :r=−0.004,p>.05 Early/preterm birth: r=0.013,p>.05	Strong
Lima et al. 1987 (43)	Colombia, Armero; 7 months	Cross-sectional; no control group; no exposure level groups	*n* = 200; mean age = 37.6y, age range 18–65 + y; F = 48%	SRQ	55% of victims were emotionally distressed. Emotional distress was associated with living alone, having lost previous job, feeling not being helped, no knowing date for leaving temporary shelter, being dissatisfied with living arrangements, complaining of non-specific physical symptoms or epigastric pain, and presenting several physical problems. Depression *p* < 0.02 Psychosomatic *p* < 0.002 Interpersonal problems *p* < 0.03	Strong
Murphy, 1984 (59)	United States, Mt. St. Helens; 11 months	Cross-sectional; control group; exposure levels group	*n* = 155; Presumed Dead Bereaved *n* = 39; mean age = 38.4y, age range 18–67y; *F* = 69.2%; Confirmed Dead Bereaved *n* = 30; mean age = 37.7y, age range 19–72y; *F* = 70%; Permanent Residence Loss *n* = 21; mean age = 42.5y, age range 30–40; *F* = 47.6%; Leisure Residence Loss *n* = 15; mean age = 54.1y, age range 33–68y; *F* = 40%; Non-Victims *n* = 50; mean age = 38.9y, age range 19–69y; *F* = 68%	LES; Hassles; SCL-90-R	Bereaved subjects reported significantly higher levels of stress and lower levels of mental health, but not physical health. Persons who lost their permanent homes reported high rates of stress, but did not report significantly higher levels of depression, somatization or poorer physical health. LES *d* effect sizes = −1.243, −0.608, −1.408, −0.261. Hassles *d* effect sizes = −0.961, −0.368, 0.001, 0.21. SCL-90-R *d* effect sizes = 0.352, 0.241, 0.111, 0.336.	Strong
Nguyen et al., 2018 (28)	Indonesia, Mt. Merapi; 7 years	Cross-sectional; no control group; no exposure levels groups	*n* = 57; age range 7–14y; *F* = 57.1%	RCPM	10.7% of the students were intellectually impaired and 96.4% showing signs of stunting.	Strong
Nzayisenga et al., 2022 (34)	Vanuatu, Ambae; 2 years	Cross-sectional; control group; no exposure levels groups	*n* = 461; mean age = 43y; F = not reported in percentages; Returned to Ambae *n* = 239; mean age = 43.7y; Displaced to Santo *n* = 201; mean age = 45.7y	IES-R	Distress was greater among woman (61.3%) but was similar on returned (58.6%) and displaced (54.7%). Professional support predicts lower distress. IES-R *d* effect size Men = 0.168 Women = 0.339	Strong
Ronan, 1997 (47)	New Zealand, Mount Reapehu; 1 year	Cross-sectional; control group; no exposure levels groups	*n* = 118; age range 7–15y; F = not reported in %; Asthma, *n* = 34; Non-Asthmatic, *n* = 79	Reaction Index; STAIC; CDI	Among children the most frequent symptom was re-experiencing (65%); 11% experienced the 3 symptom criteria: re-experiencing, hyperarousal, and numbing / avoidance; children with asthma were more likely score on high levels of hyperarousal. No effect size available.	Strong
Ruiz et al., 2014 (35)	Spain, Tagoro; 3 years	Cross-sectional; no control group; no exposure levels groups	*n* = 265; mean age = 45.43y, age range 16–over 60y; *F* = 52.5%	Place Attachment and Place Identity Scales; Scale of Perceived Restoration; PANAS Scale; Coping Strategies	Levels of fear, anger, loss and active confrontation were higher in residents nearby volcanic eruption. A greater impact on the perceived restorativeness of place and place attachment in population centres closest to the eruption. Loss *d* effect size = 1.216 Fear *d* effect size = 0.629 Anger *d* effect size = 0.721	Strong
Shore et al., 1986 (60)	United States, Mt. St. Helens; 38–42 months	Cross-sectional; control group; exposure level groups	*n* = 1,025; Low Exposure *n* = 410; mean age 45y; F = 48%; High Exposure *n* = 138; mean age 47y; *F* = 56%; Controls *n* = 477; mean age = 45y; *F* = 50%	Diagnostic Interview Schedule; SCL-90	All disaster-related onsets appeared to occur within the first 2 years following the eruption. The dose–response pattern was observed between bereaved and the property-loss groups. Men *d* = 1.445 Women *d* = 1.441	Moderate
Wakhid et al., 2022 (38)	Indonesia, Mt. Merapi; 9 years	Cross-sectional; descriptive-analytic; no control group; no exposure levels groups	*n* = 86; age range 15–64y; *F* = 40.7%	IES-R	PTSD in the mild category (72.1%); aged 26–45 years (67.4%): males (44.2%). No effect size available.	Strong
Warsini et al., 2015 (36)	Indonesia, Mt. Merapi; 2 years	Cross-sectional; control group; no exposure levels groups	*n* = 348; Cangkringan subdistrict damaged *n* = 175; mean age = 58y; *F* = 89%; Pakem subdistrict not very damaged *n* = 173; mean age = 56.3y; *F* = 51.4%	IES-R	PTSD symptoms were higher among females, aged of 18 to 59 years and people who owned their own home experienced the highest levels of psychosocial impact. No effect size available.	Strong
Zahlawi et al., 2019 (37)	Vanuatu, Ambae; 2–3 weeks	Cross-sectional; no control group; no exposure levels groups	*n* = 443; mean age = 42y; F = not reported in %	IES-R	Prevalence of high distress was 53% and was higher among woman (56%); woman who reported no available support had higher distress scores compared with other groups (average 0.37; 95% IC 0.04–0.69). No effect size available.	Strong

APAS, Abbreviated Place Attachment Scale; DASS, Depression Anxiety Stress Scale; CD-RISC, Connor-Davidson Resilience Scale; CDI, Children’s Depression Inventory; CES-D, Center for Epidemiological Studies-Depression Scale; GHQ-12, General Health Questionnaire-12-item version; GHQ-30, General Health Questionnaire-30-item version; Hassles, Hassles Scale; IES-R, Impact of Event Scale-Revised; LES, Life Experiences Survey; NEP, New Ecological Paradigm; PANAS Scale; PC-PTSD, Primary Care PTSD; PDS, Post-Traumatic Stress Scale; PSS-4, Perceived Stress Scale; PSS-SR, Post-Traumatic Stress Disorder Symptoms Scale, Self-Report version; RCPM, Raven’s Colored Progressive Matrices; SCL-90, Symptom Checklist-90; SCL-90-R, Symptom Checklist-90-Revised; SRQ, Self-Reporting Questionnaire; STAI, State–Trait Anxiety Inventory; STAIC, State–Trait Anxiety Inventory for Children; d, Cohen’s d; OR, Odds Ratio.

**Table 2:** Characteristics of the longitudinal studies included in the review.

First author, publication year	Country, volcano, period since eruption (first time)	Study design	Participants (*n*, mean age, % gender)	Follow-up		Outcomes measures	Significant findings	Study quality
				Participants (*n*, mean, age, % gender)	Duration			
Araki et al., 1998 (29)	Japan, Mt. Unzen – Fugen; 3 years	Mixed longitudinal; control group; no exposure level groups	*n* = 5,213; range age over 16y; Evacuees *n* = 4,115; Control *n* = 1,098	Not reported	4 years	GHQ-30; ICD-10 Diagnostic	67% evacuees scored markedly higher suggesting psychiatric problems. In the 4th study, 65.2% of the evacuees scored lower than at the 1^st^ screening, indicating an improvement.	Moderate
Hlodversdottir et al., 2016 (30)	Iceland, Eyjafjallajökull; 6–9 months	Longitudinal; population-based prospective cohort; control group; exposure level groups	*n* = not reported; Exposed *n* = 1,132; age range 18– ≥ 71 y; *F* = 50.9%; Non-exposed *n* = not reported	*n* = 1,180; Exposed *n* = 815 (Low exposure *n* = 90; Medium exposure *n* = 428; High exposure *n* = 267) age range 18– ≥ 71 y; *F* = 54.1%; Non-exposed *n* = 365; age range 18– ≥ 71 y; *F* = 55%	3 years	GHQ-12; PSS-4; PC-PTSD	Between 2010 and 2013 in the exposed group a decrease was found in PTSD (OR 0.33; 95% CI 0.17 to 0.61; ≅d=−0.612 ), while the prevalence of psychological distress (OR 0.74; 95% CI 0.43 to 1.27; ≅d=−0.166 ) and perceived stress (OR 0.82; 95% CI 0.50 to 1.36; ≅d=−0.109 ) remained similar. Reported multiple symptoms in 2013 were associated with perceived stress (OR 2.86; 95% CI 1.23 to 6.23; ≅d=0.579 ) and PTSD symptoms (OR 3.21; 95% CI 1.13 to 8.33; ≅d=−0.643 ), but not psychological distress.	Strong
Hlodversdottir et al., 2018 (46)	Iceland, Eyjafjallajökull; 6–9 months	Longitudinal; prospective cohort; control group; exposure level groups	*Adults:* exposed *n* = 433; age range 18–80 y; *F* = 52.7%; non-exposed *n* = 200; age range 18–80 y; F = 49% *Children:* exposed *n* = 781 (Low exposure *n* = 78; Medium exposure *n* = 485; High exposure *n* = 218); age range 0–18 y; F = 47%; non-exposed *n* = 372; age range 0–18 y; F = 50%	*n* = not reported; mean age = not reported; F = not reported; Children exposed *n* = 475; Children non-exposed *n* = not reported	3 years	GHQ-12	Exposed children compared with non-exposed children showed greater anxiety or worries (medium exposed OR 2.39; 95% CI 1.67 to 3.45; ≅d=−0.480 ) high exposed OR 2.77; 95% CI 1.81 to 4.27; ≅d=0.562 ). Children whose homes were damaged were at increased of anxiety/worries (OR 1.62; 95% CI 1.13 to 2.32; ≅d=−0.266 ) and depressed mood (OR 1.55; 95% CI 1.07 to 2.24; ≅d=0.242 )	Strong
Lima et al., 1993 (32)	Colombia, Armero; 1 year	Longitudinal; no control group; no exposure groups	*n* = 113; mean age = not reported; F = not reported	*n* = 113; mean age = 38.9y; F = 55%	5 years	SRQ	Emotional distress decreased from 65% in 1986 to 31% in 1990.	Moderate
Murphy, 1986 (33)	United States, Mt. Saint Helens; 11 months	Longitudinal; control group; exposure level groups	*n* = 155; mean age = not reported; F = not reported; Bereaved group *n* = 49; Property Loss group *n* = 18; Control group *n* = 43	*n* = 155; mean age = 34.5 y; F = 70%; Bereaved group *n* = 49; Property Loss group *n* = 18; Control group *n* = 43	3 years	LES; Hassles; SCL-90-R	Mental distress decreased between 11 to 35 months post-disaster. Mental health of the bereaved group remained poorer than both the property loss and control groups. Only 4% reported complete recovery from disaster loss after 3 years.	Strong
Ohta et al., 2003 (44)	Japan, Mt. Unzen; 6 months	Longitudinal; no control group; no exposure level groups	*n* = 248; mean age = 53.2 y, age range 19–85y; F = not reported in %	Not reported	12 months, 24 months, 44 months	GHQ-30	Psychological distress decreased from 66.1% (at 6 months) to 45.6% (at 44 months); depression began to improve only after 44 months.	Strong

GHQ-12, General Health Questionnaire-12-item version; GHQ-30, General Health Questionnaire-30-item version; Hassles, Hassles Scale; LES, Life Experiences Survey; PC-PTSD, Primary Care PTSD; PSS-4, Perceived Stress Scale; SCL-90-R, Symptom Checklist-90-R; SRQ, Self-Reporting Questionnaire; d, Cohen’s d; OR, Odds Ratio.

### 4.1 Quality assessment

The last column of Tables 1, 2 displays the quality assessment of the studies included in this review. The quality of the studies ranged from 0.68 to 1, out of a possible score of zero to 1. The level of agreement between the independent reviewers was high (*ICC* = 0.923). In general, the studies that met the selection criteria achieved reasonable quality ratings. Twenty studies were assessed as strong quality, four studies were assessed as moderate quality, and none study studies were assessed as weak quality. In the majority of the studies, the main sources of bias were related to confounding.

### 4.2 Descriptive results: studies and participants characteristics

The studies included in this review were published from 1984 to April 2023. The year with most published studies was 2022 (*n* = 3). There is a large number of years (6 years), from 1987 to 1993 and from 2006 to 2012, without studies that met the inclusion criteria. The journal with the largest number of articles published was BMJ OPEN, which had three studies. In general, the journals had just one published article (*n* = 17). The majority of studies were conducted in Iceland (*n* = 5) and Japan (*n* = 5), followed by Indonesia (*n* = 4) and the United States (*n* = 3). Fewer studies were conducted in Vanuatu (*n* = 2), Spain (*n* = 2), Colombia (*n* = 2) and New Zealand (*n* = 1). The data showed representation of Europe, Asia, Oceania and America, but there is currently a lack of studies from Africa. Volcanoes that generated the largest number of studies were Eyjafjallajökull in Iceland (*n* = 5), and Mount Merapi in Indonesia and Mount Saint Helens in the United States, both with three articles.

Of the eligible 24 studies, 18 were cross-sectional, and 6 were longitudinal. Specifically, population-based/ cohort designs in both cases were the most frequent (*n* = 5). The longest period from the volcanic eruption to the conduction of the study was 9 years, and the shortest 2–3 weeks. In longitudinal studies, the most frequent follow-up time was 6–9 months, ranging from 6 months to 3 years. The most commonly used questionnaires to assess mental health were the Post-Traumatic Stress Diagnostic Scale (PDS) with 6 studies, and The Impact of Event Scale-Revised (IES-R) with 4 studies.

The sample size of the studies ranged from 56 (28) to 5,213 (29). The total number of participants in the included studies was 17,166, of which 1,249 were children. Gender and age were reported in 23 of the 24 studies. In those studies that clearly recorded the gender of the participants, 9 of the studies (28, 30–37) reported a slight female predominance, in comparison with males with six studies (38–43). In this review, data were encoded in children and adolescents ≥ 17, adults ≤ 18 and older adults over 65 years of age. The age range was from 0 (30) to 85 years of age (44). There were four studies that specifically evaluated children and adolescents (28, 45–47), while the participants of 3 studies (29, 35, 38) were included in the category of adults despite including adolescents. Twenty–two studies evaluated adults, of which only 10 (30, 40–46, 48, 49) listed adults over 65 years grouped into separate categories of young adults and middle-aged adults. However, the studies reported different coding in the age ranges, especially in adults [e.g., Kamijo et al. (42); Carlsen et al. (45)], implying that data could not be obtained for an intergenerational comparison.

### 4.3 Preliminary considerations

We identified possible conceptualization problems of reported psychological disorders. Therefore, we considered it appropriate to provide a brief definition of the most prevalent disorders reported in the studies, based on the general literature of the field of psychology, to facilitate a common framework. For example, we found that the studies included in this review used a wide variety of terms to refer to distress symptoms. According to the Diagnostic and Statistical Manual of Mental Disorders, Fifth Edition (DSM-5), distress is significantly associated with mental disorders, but the Handbook did not provide a conceptual framework on the precise nature of distress (50). The DSM-5 (51) proposes psychological distress as a technical term to define “a range of symptoms and experiences of a person’s internal life that are commonly held to be troubling, confusing, or out of the ordinary.” In the literature, the term psychological distress is the most commonly used to refer to non-specific symptoms of stress, anxiety and depression (52). Post-traumatic stress disorder is the most prevalent psychological disorder after exposure to a traumatic event. However, the current definition of the disorder remains controversial (53). DSM-5 indicates that the development of PTSD requires exposure to one or more traumatic events, and whose central symptoms concern intrusions about and avoidance of memories associated with the traumatic event itself (51). In contrast to PTSD, depression may or may not be associated with a traumatic event (51). The most common symptoms of depression are anhedonia, feelings of worthlessness, sleep disturbances, concentration difficulties, and suicidal ideation (54). Stress is defined by the DMS-5 (51) as a person’s specific and nonspecific response pattern to an event that exceeds their ability to cope with it. The term stress is usually a symptom not specifically used in many disorders and questionnaires such as the DASS (55), in which stress corresponds to symptoms of irritability, tension and agitation (56). However, a recent study urges caution in understanding stress as a universal mechanism that exists across time and cultures (57). Anxiety disorders include disorders that share features of symptoms that include worry, social and performance fears, unexpected and/or triggered panic attacks, anticipatory anxiety, and avoidance behaviours (58). In particular, individuals diagnosed with generalized anxiety disorder have persistent and excessive anxiety and worries about several domains that are difficult to control (51).

### 4.4 Outcomes characteristics

In this review, a small subset of psychological disorders investigated among the exposed population were found to recur in the majority of studies, compared to other disorders whose presence was anecdotal. In the articles in this review, the most studied psychological disorders were psychological distress with nine studies (30, 32, 34, 37, 43–46, 49), and PTSD with also nine studies (30, 36, 38, 40–42, 45, 47, 49), followed by stress with seven studies (30, 31, 33, 39, 45, 49, 59), depression also with seven studies (33, 40, 41, 44, 45, 47, 59), and anxiety with three studies (39, 45, 47).

Of these 24 studies, there are a few studies that have their particular conceptualizations: one study explored the relationship between seeking professional help in people with mental disorders (40); another study examined the influence of professional support on the development of mental disorders (34); one study associates severity of psychological disorder to certain jobs such as being a police officer (42) and finally, another study investigated the stress experienced during the pregnancy of their mothers in newborn’s (31). The exploration of psychological symptomatology in children and adolescents appears in 7 articles, but only 3 (28, 46, 47) focused entirely on children. Two studies assessed child behaviour (45, 46), and in one study, the researchers registered the assessment from the paternal perspective of the symptoms exhibited by their children (46). Another article compared the differences between children with asthma and those without asthma in the influence of psychological symptoms (47).

Results indicate that 87.05% (*n* = 21) of the studies included in this review can be categorized as studies that measured psychological disorders in populations exposed to volcanic eruptions. However, despite assessing psychological outcomes, the study of Escolà-Gascón et al. (39) fit better in the next section due to their relationship with other factors. Fifteen of the 23 studies included in this review used cross-sectional designs (31, 34, 36–38, 40–43, 45, 47–49, 59). Seven studies had only an experimental group or exposed population group (37, 38, 40–43, 45), and 8 studies had at least one non-exposed group that could be considered equivalent to a control group (31, 34, 36, 47–49, 59, 60). Of the studies with control groups, 2 present a subdivision of the level of exposure of the populations due to the proximity to the volcano (48, 49), and 2 assessed the level of exposure according to the degree of loss of property or death of a family member (59, 60). Six of the included studies had longitudinal designs (29, 30, 32, 33, 44, 46). Two of these studies had only an exposed group, with no control group (32, 44), and four of the studies had at least a non-exposed group that can be considered as a control group (29, 30, 33, 46). Finally, two studies with control groups, were divided into different subgroups according to the level of exposure in terms of the proximity of the populations exposed to the volcano (30, 46) and one due to the loss of property or death of family members (33).

Of the included studies, only two studies (9.09%) investigated how mental health is related to emotions and a series of environmental factors (35, 39). Both studies were cross–sectional studies and heterogeneous in terms of the variables assessed. In this review, we found no studies that assessed eco-emotions. Some studies cited above refer to the assessment of emotions and environmental factors (45, 46, 49), but the main topic investigated was not the same.

Only 4.17% (*n* = 1) assessed cognitive functions in populations exposed to volcanic eruptions (28). It was a cross–sectional study without a control group and measured the prevalence of intellectual impairment among children and adolescents.

### 4.5 Results of the cross-sectional studies

#### 4.5.1 Psychological disorders

The data provided by the retrieved studies showed that the most prevalent psychological disorder among the population exposed to the volcanic eruption was psychological distress, but this review also found a wide range of incidence of this disorder, between 10 and 60% (30, 34, 37, 45, 49), followed by depression between 8 and 30% (38, 45), anxiety and stress disorders with percentages with 7–8% (45), and PTSD, around 7% (30, 45, 49).

In exposed populations, being female was a significant factor in the susceptibility to developing PTSD (36, 42, 45, 49), depression (41, 45), psychological distress (34, 37, 45), stress (31, 45) and anxiety (45). Regarding age, young and middle-aged adults between 18 and 59 years old have a higher risk of showing PTSD (36, 38, 49), psychological distress (45, 49), depression, stress and anxiety (45). Studies also suggest that having less education may be a predictor of suffering PTSD (41, 49). In contrast, having a better education was associated with higher levels of psychological distress (37, 49). Similarly, being widowed or divorced [e.g., Goto et al. (41)] predisposed to the development of PTSD, but there was an increased risk to suffer of psychological distress in people who were married or in a relationship (49).

According to exposure levels to volcanoes, those who pertained to the most exposed groups had a higher probability of increasing the symptoms of psychological distress (49), PTSD (36, 49), and psychological morbidity (48). However, the studies did not report differences in perceived stress between non-, low- and high-exposed groups (49).

The results demonstrated that several factors significantly influenced vulnerability to developing the most prevalent psychological disorders. In this review, we have placed them in the category of predictors of severity. For example, the loss of a significant person during the volcanic eruption was linked to high levels of stress and low levels of mental health (59). Also, the loss of a pet was associated with high scores in PTSD symptoms and depression (41). In this line, those affected by the volcanic eruption who owned their homes had higher risks of developing PTSD than those who lived in rented houses (36). Likewise, loss of property or uncertainty of loss of home were indicative of risk factors for developing PTSD symptoms (41), and stress (59). Evacuation appeared as a risk factor for suffering anxiety, depression, and stress (45). The number of previous experiences of disaster evacuation presented unclear results since it is suggested as a vulnerability factor for depression (41), but familiarity with suffering a disaster might have some protective effect on PTSD (36). Findings showed that in pregnant women the stress related to evacuation was associated with decreasing birth length (31). In contrast, psychological distress showed a similar prevalence between displaced and returnees (34), even indicating that the participants who did not live in the evacuated area had higher levels of distress (45). The availability of psychological support also showed contradictory effects. For example, a study did not find differences between levels of distress and the use of professional support (34). In contrast, another study reported that women that did not have availability of support had higher levels of mental distress (37). Findings also showed that the severity of PTSD and depression symptoms were positively correlated with help-seeking from physicians, but no psychologist or mental health professionals (40). A risk factor often underrated is being a professional helper in an emergency crisis. In this review, we found that being a police officer on duty during the volcanic eruption was associated with PTSD symptoms, with severity factors such as more than seven cumulative days at work and selecting drinking and/or smoking as stress relief after a disaster–support work (42).

PTSD symptoms and depression were highly correlated, with a comorbidity of 10.7% (38). Emotional distress was significantly related to complaints of epigastric pain, non-specific symptoms and an increasing number of physical complaints (43). Also, having one or more symptoms from the nose, eyes, or upper respiratory tract was associated with psychological morbidity (48).

Some studies investigated PTSD, anxiety/worries and behavioural problems in children (45, 47). These studies found that in this population, the most frequent symptom of PTSD was re-experiencing the experience of the eruption and that children who met symptoms of PTSD also manifested clinically significant levels of anxiety and depression (47). Children with asthma had more symptoms during the ash fall (45), and a significant high score in hyperarousal levels was more likely than in children who did not have asthma (47). Other symptoms reported in this group during the eruption were headaches, nausea or stomach pain, and sleep-related problems (45).

In older adults, several studies suggested that older evacuees are more vulnerable to developing PTSD (40, 41) and distress (43), but another found that older adults (over 60 years) had a lower risk of developing PTSD (36). Among women, age was negatively associated with distress (37). In addition, older adults tended to seek more professional help from primary care physicians and social workers than younger adults (40).

#### 4.5.2 Emotions, eco-emotions, and environmental factors

The results showed a higher number of negative emotions, such as fear, anger, and loss, among residents living near the volcano, compared to positive emotions, with interest as the only significant emotion. Likewise, the inhabitants of the place closest to the volcanic process presented an increase and intensity in these negative emotions, which was not found in the positive emotions. However, they felt each of the four emotions equally, in contrast to the lowest exposed inhabitants. In the most highly exposed population, significant use of active coping and analysis strategies was found, indicating more adaptation, while the situation in the lowest exposed populations was the opposite, scoring higher in denial (35). According to the sense of place, measured from dimensions of place identity and place attachment, it tends to decrease systematically at the onset of a volcanic eruption and continues to decrease after the remission of volcanic activity (39). Ruiz et al. found that attachment to the place decreased only in highly exposed populations and that this decrease was significantly explained by the emotion of loss, but, contrarily, place identity was not affected in any of the areas during the eruption. Findings showed that, among those affected by the volcanic eruption, there was a reduction in pro-ecological attitudes or beliefs, indicating a conflict with the environment and lower attitudes in favour of environmentalism. This trend stabilized 2 months after the natural disaster occurred (39). Similarly, in relation to perceived restorativeness, a significant reduction in the positive perception of the environment was found only in the areas closest to the volcano (35).

#### 4.5.3 Cognitive functions

In this review, we found just one article that assessed cognitive functions in populations exposed to volcanic eruptions. Specifically, among exposed children and adolescents, 10.7% had impaired intelligence, but no direct causes were found in the study. Drinking water analysis was carried out to determine contaminants, and also all the chemicals detected were in safe ranges. The authors argue that malnutrition and poverty might be underlying these cognitive impairments since 96.4% of the sample were shorter and 14.3% were underweight for WHO’s standard (28).

### 4.6 Results of the longitudinal studies

The main findings reported in longitudinal studies indicated that distress (32, 33, 44), depression (44), PTSD and perceived stress (30) in the exposed population progressively decreased with time. However, the pattern of decline was not the same across the different psychological disorders. In some cases, the results were contradictory or insufficient. For example, psychological distress decreased between 35 months (33), 44 months (44) and 4 years (32) after the eruption. Depression showed delayed improvement and only improved after 44 months (44). A lower prevalence was found for PTSD symptoms 3 years later in the exposed population (30). Despite this decline over time, only 6% of the participants reported complete recovery after 30–35 months from disaster (33).

Considering the levels of exposure to the eruption and whether this affects the presence of psychological symptoms for a more extended period, the prevalence of perceived stress remained similar in the exposed regions, but PTSD symptoms were only found in the medium and high exposure groups 3 years later (30). In contrast, psychological distress had unclear results because Hlodversdottir et al.’s (30) study did not find significant differences between exposed groups, while Murphy’s (33) indicated that the bereaved group (high exposure) remained with significantly higher scores than the rest of the groups.

The most important factor in the severity of psychological disorders for the exposed population was to remain evacuated. In particular, psychological distress still scored at 46% among evacuees after 44 months (44). The predictors of emotional distress 3 years later were surprisingly the most infrequent symptoms such as unhappiness, daily work suffering, feeling unable to play a useful part in life, and feeling useless (32). In addition, 3 years later, having multiple symptoms (morning winter phlegm, nocturnal or daytime winter phlegm and/or chronic nocturnal or daytime winter phlegm and skin rash/eczema) were associated with perceived stress and PTSD symptoms but not psychological distress (30).

Findings in age groups showed that in highly exposed children, both genders had an increased risk of suffering anxiety/worry symptoms, and no significant decrease of symptoms was detected in 3 years. Parents with symptoms of psychological morbidity reported the same prevalence of symptoms among their children as other parents; although not statistically significant, a tendency was observed for increased anxiety/worries and headaches (46). In older adults, the symptoms were more persistent (44 months) over time compared to younger adults (24 months), and they showed a predisposition to develop psychological distress (44).

## 5 Discussion

This systematic review aimed to examine the effects of volcanic eruptions on the mental health and cognition of people of all ages who are exposed to them. We reviewed the results of 24 studies published from inception to 2023. In the analysis of the results, we identified three well-differentiated themes, which we have considered as psychological disorders, emotions, eco-emotions and factors related to the environment, and cognitive functions. In addition, data on prevalence, severity factors, comorbidity, and vulnerable populations were extracted from the studies. The main findings of the cross–sectional and longitudinal studies on exposed populations are discussed below.

### 5.1 Cross-sectional studies

Three main themes emerged in the cross–sectional studies included in this systematic review. Psychological distress was the most studied and the most prevalent among the psychological disorders observed in the exposed population with a wide range of incidence comprising 10–60% (30, 34, 37, 45, 49). This was expected because the term distress included a wide variety of psychological symptoms and, in most cases, is considered a general measure and not a specific mental health outcome. Carlsen et al. (45) found that 39% of the 207 participants living close to the Eyjafjallajökull volcanic eruption showed symptoms of mental distress a few weeks after the eruption that were associated with feeling helpless or being awakened by noises from the volcano. Warsini et al. (36) investigated the impact of the Mount Merapi eruption and found that survivors remained traumatized 2 years after the eruption. The study showed that a key factor in developing PTDS in survivors was familiarity with the disaster.

Females were the most vulnerable population. In most studies, females scored higher than males in all psychological disorders (31, 34, 36, 37, 42, 45, 49), especially in psychological distress, which reached 49% (45). Women reported more frequent mental health problems than men, with the highest rates in those between 35 and 49 years of age. In Warsini et al. (36) study, female eruption survivors had a higher risk of developing PTDS than male survivors. Available support was important. Distress scores were higher among women than men, with higher scores among women without available support (37). These findings support the importance of psychosocial support in natural disasters.

The results did not provide a clear answer to whether young adults, middle-aged adults, or older adults were more predisposed to develop psychological disorders. While Warsini et al. (36) informed that survivors over 60 years of age had a lower risk of developing PTDS compared with younger and middle-aged adults, Wakhid et al. (38) found increased risk among young adults and middle-aged adults (26–46 years of age) and Goto et al. (41), reported more severe PTDS symptoms in older adult participants, Several reasons may explain why older adults in the Warsini et al. study were more resilient than the younger adults, including that older adults are more likely to have experience coping with traumatic life events, and had suffered previous natural disasters that made them more resilient. Young adults have more to lose from the eruption than older adults, which may produce more stress. The exposure level can also influence the age range that develops a certain psychological disorder. Gissurardóttir et al. (49) found that exposure level was a risk factor for psychological distress for young and middle-aged adults (18–50 years old) but not for older people (51–80 years old). In children, it was found that exposed children, compared to non-exposed children, had more worries, anxiety and behavioural problems. This may be derived from the need to stay inside for 6 days or more to avoid volcanic ash (45).

Special consideration should be given to children and older adults in the exposed population. The groups that were more exposed to volcanic activity than those that were less or non-exposed showed higher levels of severity in the symptomatology, both in children and adults. Likewise, there are predictors of severity that were related to an increase in psychological disorders, in particular, the effects of loss and evacuation (36, 49). Loss was understood as a broad concept encompassing family members, pets and personal property. Evacuation and/or relocation were closely related to the duration of evacuation and whether the people had already experienced previous evacuations. The relationship between loss and the severity of PTSD symptoms was suggested (36, 41). Similarly, the severity of the evacuation and/or relocation was closely related to the duration of the evacuation and whether the evacuees had already experienced previous evacuations. The results suggest that evacuation is linked to depression (41, 45). In contrast, psychological distress showed similar scores among displaced and returnees (34). The availability of psychological support was another factor but results were contradictory. Psychological support was especially significant for psychological distress (34, 37), but no for PTSD and depression (40), whose severity symptoms not correlated with seek-help professional mental health support. In Goto et al. (40) study the findings might be influenced by culture and might not generalize.

Among exposed populations, reporting two or more respiratory symptoms due to volcanic ash was quite common, and this was linked with the prevalence of psychological morbidity (48). In contrast, emotional distress was more related to complaints of epigastric pain and non-specific symptoms (43). These results indicate that those with many symptoms represent a more sensitive subgroup within the exposed population, which should be especially targeted (48).

The studies in the field of emotions and factors related to the environment, although few, are important because they showed a new approach from environmental psychology and ecopsychology to the mental health consequences in populations exposed to volcanoes, especially relevant in the context of climate change. In this review, it was found that volcanic eruptions increase negative emotions and produce a reduction in pro-ecological attitudes among the exposed population. However, an interesting result is that the two most intense emotions were “interest” (a positive emotion) and “loss” (a negative emotion), which may show how the population experienced the volcanic eruption (35). Another study reported a reduction in pro-ecological attitudes in the exposed population, supporting the theory that the victims of the volcanic eruptions conflict with the environment, and experience a decrease in the sense of place which indicated a process of detachment with the environment (39).

Exposed children in the study by Nguyen et al. (28) showed intelligence impairments, but no direct causes were found. Among the possible causes the analysis of drinking water did not find high levels of chemical concentrations, but the effects of malnutrition might contribute to the high prevalence of intellectual impairments among exposed children, as the authors explained (28).

### 5.2 Longitudinal studies

In the longitudinal studies, only the theme of psychological disorder was found, and therefore, no results were obtained on emotions and factors related to the environment or cognitive functions.

An important finding is that the psychological disorders produced by the eruption are long-lasting and persist over time, being decrease not progressive in all cases. However, an overall improvement was found in PTSD, depression, and distress 3 years after the eruption (30, 44). Ohta et al. (44) reported that most parameters of psychological distress in evacuees improved from 6 months to 44 months after the evacuation due to the volcanic eruption. Psychological distress decreased from 66.1% at 6 months to 45.6% at 44 months. The authors pointed out that the score was very high compared to other studies conducted among the general population. The results suggest a clear persistence of psychological distress among the eruption victims. In contrast to distress, depression only began to improve after 44 months of the eruption or no improvement at all. It is important to note that the assessment was related to the deterioration of interpersonal relations. The recovery from distress was more difficult in victims over 50 years of age and older adults. These persons are more vulnerable as they need to rebuild their lives after the disaster. Murphy’s (33) longitudinal data collected at 1 and 3 years, post-disaster in three groups (bereaved, loss and control) found significant differences between the loss and control groups on stress and mental health results. Although the scores of the bereaved group decreased with the passage of time they were higher than the control group in several mental health outcomes, and significantly higher than the property loss group. The results suggest that victims of volcanic eruption have long and difficult recoveries due to many different factors, including timing in the life cycle, lack of control, and difficulty in finding meaning to the tragedy, among others.

The results of the longitudinal studies are similar to those of the cross-sectional studies. Especially exposed populations had a higher prevalence of developing psychological disorders, and it increases according to the exposure level to volcanic activity. However, some inconsistencies were found in the prevalence over time of psychological disorders, particularly among evacuees. In Ohta et al. (44), a longitudinal study conducted over 44 months, the prevalence of psychological distress among people who remained evacuated from the disaster area was persistent, at high rates, throughout the study. In contrast, in Nzayisenga et al. (34), a cross-sectional study conducted 2 years post-displacement reported a similar prevalence of distress among displaced and returnees, in both cases with high scores. This finding may be explained by the fact that Ohta et al. (44) did not have a control group to compare with, and in Nzayisenga et al. (34), the group of returnees was also displaced for a time. The longitudinal studies included in this review suggest that the improvement of psychological disorders was associated with a reduction in volcanic activity and its aftermaths for the exposed population (29).

### 5.3 A special case: the Mount Saint Helens disorders

A special case was the so-called Mount Saint Helens Disorders (MHS-D) described by Shore and colleagues (60). MHS-D was related to those affected by the Mount Saint Helens volcano. This disorder was composed of three psychological factors: depression, generalized anxiety and post-traumatic stress. Individuals who suffered property loss or the death of a family member or close relative were defined as the high-exposure group (60). In this study, the MSH-D had a prevalence of ratios in the high exposure group of 11.1% in men and in women of 20.9%. In the high-exposure group, the dose–response pattern occurred among both the bereaved and the property–loss victims. The rate of onset in the high-exposure group dropped sharply, and 2 years following the eruption, no new cases were observed in this group.

### 5.4 Other findings: eruption experiences

Some studies included in this systematic review investigated how certain environmental factors related to the experience of volcanic activity (e.g., noise, earthquakes, volcanic ash, etc.) influenced the development of psychological disorders. Residents in exposed areas had an increased risk of developing PTSD, psychological distress, and perceived stress due to experiencing directly the consequences of volcanic activity in their daily lives, such as material damages, staying outside during ashfall for work, or having to wear protective equipment because they were outside (49). Depression and stress were more common among those who had experienced earthquakes, and psychological distress was significantly higher in those who had been awakened by the noise of the eruption (45). Likewise, the symptoms of PTSD, mental distress, and perceived stress developed when having a permanent view of the volcano from their home or there was a feeling of insecurity (49). Feelings of helplessness and being afraid were common factors for the onset of PTSD, psychological distress, depression, and anxiety. In addition, thinking that one’s life was in danger was associated with PTSD and depression, while thinking that someone’s life was in danger was related to the presence of PTSD and stress (45). Children whose homes were damaged by the eruption had increased anxiety/worries and depressive mood (46). Furthermore, staying indoors during ashfall has been found to be a risk factor for the development of mental distress and behavioural problems in children (45).

## 6 Strengths and limitations of the review

The current review has several strengths. To the best of our knowledge, this is the first systematic review exploring the effects on the mental health of populations who have experienced or are currently experiencing a volcanic eruption or its aftereffects. Likewise, an evaluation of the quality of the studies was conducted using Standard Quality Assessment Criteria for Evaluating Primary Research Papers from a Variety of Fields by Kmet et al. (26). See Supplementary Material S1.

This review, however, is not without limitations. A limitation of the present review is not having included qualitative studies. The exclusion of qualitative studies may limit the understanding of individual’s experiences and perceptions influencing mental health outcomes. By considering only peer-reviewed journals, potential research published in grey literature or conference proceedings may have been missed. The fact that this review included only articles published in English might be a limitation. However, most studies are published in English, the common language for scientific publications. Therefore, we hope that the most relevant studies on the subject have been included in the review. Although eligibility criteria were previously established, some discussion and consensus building among the authors was needed to define the dimensions of mental health and when a population was considered to have experienced a volcanic eruption first-hand. Additionally, since the geographic distribution of each article was determined by its respective volcano or volcano location, this may have resulted in some unintentional errors in extracting the data. The results obtained in the review were based on a limited number of studies and their scope. Some of the limitations were identified by the authors of the studies included in this review, such as the heterogeneity of the samples, a wide variety in terms of study design, primary purpose, and outcome measures. In many studies, these factors were related to the singularity of the volcanic eruption, such as the type of volcano, the duration of the eruption, its localization close to towns, evacuation, etc., but these were not sufficiently described. This translates into limitations in the ability to compare findings across studies. Finally, we have not published or registered the protocol of this review.

## 7 Conclusion

The results of this systematic review suggest that among exposed populations of all ages, volcanic eruptions produced several psychological disorders with scores that can be considered clinically significant, and this risk increases according to the proximity of the place of residence to the volcano. Likewise, those studies that explored negative emotions and pro-ecological attitudes are especially noteworthy for their novelty. Unfortunately, there were not enough studies on cognitive functions in the exposed population to provide significant and consistent results.

In this review, we identified that research on mental health in the population exposed to volcanic eruptions focused mostly on a very small subset of the psychological disorders possible in these individuals and ignored the measure of other significant disorders that might be also present [e.g., schizophrenia (61), phobias and psychotic disorders (62), etc.]. Moreover, certain studies [e.g., Zahlawi et al. (37)] did not take into account the diagnosis of acute stress disorder during the first month after exposure. In fact, the diagnosis of PTSD should be considered when symptoms occur for more than 1 month after trauma exposure to minimize pathologizing of the normal stress reaction (51, 53). We also noted that an important aspect of the severity of the symptoms was closely related to the level of exposure to the volcanic activity and aftermaths. However, there was no consensus in the studies on the conceptualization of exposure levels. While some authors used the proximity of the exposed population to the volcanic eruption [e.g., Carlsen et al. (48)], others applied different variables for each group, for example the magnitudes of loss such as bereaved or property loss [e.g., Murphy (33)]. Another problem encountered was the mental health instruments used to assess the psychological disorders of the exposed populations. For example, in IER-S, an instrument that measures post-traumatic stress, the authors sometimes modify their instructions to measure psychological distress (37) or stress related to evacuation (31), introducing a bias effect on the results.

In summary, this review provides valuable information on how volcanic eruptions affect the mental health of the population exposed to this environmental phenomenon. However, further research is highly needed in this area using robust experimental designs and liable and well-validated psychological instruments to assess the outcomes. The assessment of cognitive processes, including selective and sustained attention, different types of memory, and executive functions, is also an important topic for future research conducted to investigate the psychological effects of volcanic eruptions on populations exposed to volcanic eruptions.

## 8 Recommendations for future research

The results of this review shed light on the relevance of further research on mental health in populations exposed to volcanic eruptions of special needs is to focus on the most vulnerable populations such as women, children and older adults, as well as in emergency responders or volunteers who might develop symptoms doing their duty. It is also important to emphasize the investigation of the possible relationship between the characteristics of the volcanic eruption, for instance, the chronicity or the type of volcano, as predictors of the severity of the symptomatology. We noticed that there is a lack of studies conducted to investigate cognitive alterations occurring in important specific cognitive processes such as attention, executive and memory functions associated with the experience of volcanic eruptions or studies investigating ecological emotions and pro-environmental behaviours, approaches that have occurred in another natural disaster. Finally, well-designed studies are needed to investigate the impact of volcanic eruptions on the mental health of the exposed populations and the development of effective interventions to overcome the psychological disorders caused by this natural disaster. In sum, it is necessary to develop specific protocols, valid and liable assessment instruments, and psychological interventions that allow to manage appropriately the mental and psychological disorders produced by volcanic eruptions.

## Data Availability

The original contributions presented in the study are included in the article/Supplementary material, further inquiries can be directed to the corresponding author/s.
