# Supplementary material for: Effects of volcanic eruptions on the mental health of exposed populations: a systematic review
Source: Front Public Health. 2024 Dec 12;12:1475459. doi: 10.3389/fpubh.2024.1475459 (PMC11672347; doi:10.3389/fpubh.2024.1475459)
Supplement: Supplementary file 1 [file Data_Sheet_1.PDF]

Supplementary Material S1.

Study	C1	C2	C3	C4	C5	C6	C7	C8	C9	C10	C11	C12	C13	C14	Mean	Rating
Araki et al. (29)	1	2	2	1	N/A	N/A	N/A	1	2	1	1	1	2	2	0.727	Moderate
Carlsen, Hauksdottir et al. (48)	2	2	2	2	N/A	N/A	N/A	2	2	2	2	1	2	2	0.954	Strong
Carlsen, Gislason et al. (45)	2	2	1	2	N/A	N/A	N/A	2	2	1	2	1	2	2	0.954	Strong
Escolà-Gascón et al. (39)	2	2	1	1	N/A	N/A	N/A	2	2	2	2	2	2	2	0.909	Strong
Gissurardóttir et al. (49)	2	2	2	2	N/A	N/A	N/A	2	2	2	2	1	2	2	0.954	Strong
Goto et al. (40)	2	1	2	2	N/A	N/A	N/A	2	2	1	2	1	2	2	0.863	Strong
Goto et al. (41)	2	1	1	2	N/A	N/A	N/A	2	2	1	2	1	2	2	0.818	Strong
Hlodversdottir et al. (30)	1	2	2	2	N/A	N/A	N/A	2	1	2	2	1	2	2	0.818	Strong

Hlodversdottir et al. (46)	2	2	2	2	N/A	N/A	N/A	2	2	1	2	1	2	2	0.909	Strong
Kamijo et al. (42)	1	1	2	2	N/A	N/A	N/A	2	2	1	2	1	2	2	0.818	Strong
Kushnick et al. (31)	2	1	2	2	N/A	N/A	N/A	2	2	2	1	1	1	2	0.818	Strong
Lima et al. (43)	1	1	1	2	N/A	N/A	N/A	2	2	1	1	1	2	2	0.727	Moderate
Lima et al. (32)	2	2	1	1	N/A	N/A	N/A	1	2	1	1	1	2	2	0.727	Moderate
Murphy (50)	2	2	2	2	N/A	N/A	N/A	2	2	2	2	1	2	2	0.954	Strong
Murphy (33)	1	2	2	1	N/A	N/A	N/A	2	2	1	2	1	2	2	0.818	Strong
Nguyen et al. (28)	2	2	2	2	N/A	N/A	N/A	2	2	2	2	1	2	2	0.954	Strong
Nzayisenga et al. (34)	2	2	2	2	N/A	N/A	N/A	2	2	2	2	1	2	2	0.954	Strong
Ohta et al. (44)	1	2	2	1	N/A	N/A	N/A	2	2	2	2	1	1	2	0.818	Strong

Ronan (47)	2	2	2	2	N/A	N/A	N/A	2	1	1	1	1	2	2	0.818	Strong
Ruiz et al. (35)	1	2	2	1	N/A	N/A	N/A	2	2	2	2	1	2	2	0.863	Strong
Shore et al. (51)	1	1	2	2	N/A	N/A	N/A	2	2	1	1	1	1	2	0.727	Moderate
Wakhid et al. (38)	1	2	2	2	N/A	N/A	N/A	2	2	1	2	1	1	2	0.863	Strong
Warsini et al. (36)	2	2	2	2	N/A	N/A	N/A	2	2	2	2	1	2	2	0.909	Strong
Zahlawi et al. (37)	2	2	2	2	N/A	N/A	N/A	2	2	2	2	1	1	2	0.909	Strong

Note: Not Applicable = N/A; Yes = 2, Partial = 1, No = 0; Quality scores: Strong = ≥75%, Moderate = 55 ≥75%, Weak = ≤ 55%
